# A Treatise on the Practice of Surgery

**Published:** 1857-01

**Authors:** 


ART. II.—*A Treatise on the Practice of Surgery.* By HENRY H. SMITH, M.D., Professor of the Principles and Practice of Surgery in the University of Pennsylvania; Consulting-Surgeon to the St. Joseph's Hospital, Philadelphia. Author of a Treatise on Operative Surgery, etc. etc., illustrated by two hundred and seventy-four engravings on wood. 8vo. pp. 828. Philadelphia. J. B. Lippincott & Co.

A satisfactory text-book on surgery remains to be written. Perhaps the thing itself is impossible. Limited space, the stern necessities of a single octavo, which must contain a compend of every surgical fact or theory, practical or pathological, forbid amplification and cramp the efforts of the impatient writer. *Nolens volens,* he must repeat that never-to-be-sufficiently-enunciated fact, that heat, pain, redness, and swelling, are the four cardinal points of inflammation; enlarge upon the goodness of that Providence which incloses an abscess in a membrane, and leads its pus toward the surface; and define again, for the five hundredth time, the distinction between gangrene and mortification. All this must be done because we, the reading medical public, have said so; we have virtually decided that all books, not devoted to specialties, shall be written down to the comprehension of the first-course student; and that the intelligent practitioner, who has been duly posted on these stale matters of fact by every surgical work in his library, shall renew his early teachings by a reperusal, and be left to seek the new things of science among the overshadowing mass of old.

He will be a bold author, and, moreover, one utterly regardless of pecuniary results, who shall strike out a new course, and write a book made up of positive additions to science. Such a volume may not attain to the dignity of octavo dimensions, but what a treasure would even a duodecimo be to the practitioner, wearied with this endless reproduction of the rudiments.

That this repetition is a fault chargeable upon Professor Smith's new work, is no very serious accusation. The author, in his preface, speaks of it as a text-book, intended to assist the students of his class. That its scientific qualities are sufficient for their end no one will deny, and we shall possibly find that it possesses a few higher excellencies. We may here say, however, that so long as medical authorship is made a mere waiter and helper in the teaching of under-graduates, so long as men write because they are professors, and not because they have something to tell, just so long will our literature partake of the school-boy character, the lack of scientific accuracy and precision, which is the bane of our medical writings.

The term "Practice of Surgery," is somewhat indefinite. Practical surgery would include all surgical operations and treatment, leaving entirely out of view the theories, pathological or therapeutical, on which those operations are based. In the sense employed by Professor Smith, it includes far more than the mere practice of the art; and embraces surgical semeiology and pathology. He seems to have lost the distinction between principles and practice, and, as a result, we suffer from a confusion of ideas as to the light in which it is intended to be viewed.

To come at once to particulars, we may remark, that the opening chapter on "Surgical Diagnosis," is a systematic and careful *resumé* of the general principles which govern the recognition of disease. We appreciate the evidences of system which this chapter displays, and though there is nothing original in the idea that certain signs are common to diseases of certain tendencies, and that much may be gained by a careful inspection of the posture, physiognomy, color, temperature, and sensations of the patient, yet the text-book which failed to impress this important fact upon the student, would be deficient in an essential particular. We have seen many an unfavorable prognosis formed on the simple fact that the patient "looked badly," and too often have witnessed the verification of that prognosis. A full analysis of "characteristic symptoms" is a desideratum. The task would be an interesting one, but would require far more than ordinary attainment. A profound knowledge of those principles of physiology, which underlie all true pathology is the first requisite; with it, must be coupled an intimate acquaintance with the physiognomy of disease.

A brief chapter "On the Use of the Senses in Forming a Diagnosis," strikes us as an excess of refinement. It is one of those hair-splitting, intensely logical distinctions, which ordinary mortals fail to appreciate. How, we may ask, is the surgeon to form a diagnosis *without* the use of the senses? Deaf, dumb, blind, tasteless, incapable of smell; a diagnosis is an impossibility. The shrewd surgeon need not be told that he is to recognize a change in color, by the eye; and that it will then be very well for him to feel of it, and ascertain its hardness or softness. There is a lack of directness in all such fine-drawn notions, and, though their topic is faultless, their utility is not beyond question. Fortunately, this chapter, into which a love of system has betrayed Professor Smith, is a brief one; had it been omitted altogether, our ideas of propriety would have been better pleased.

"Surgical pathology," we are told in the beginning of Part II, "as a branch of general pathology, embraces the considerations of the characters and *means of relieving* external disorders." The looseness of definition in the words we have italicized, should be corrected in any subsequent edition. The "means of relieving" diseased action are no part of pathology.

In the discussion of "inflammation," the ancient division of its causes into exciting, predisposing, and essential or proximate, is adopted. Perhaps no form of classification has been more fruitful in confusion than this. We may speak appropriately of an injury as an exciting cause; a predisposing cause is no cause at all, but simply a condition of the system, liable to be affected by an exciting cause; while in all our theories of "error loci," "lentor," or spasm of the capillaries, it is evident that the *actions* of inflammation are confounded with its *causes:* that is, our proximate causes are simply a part of the process. A dispute is never so interminable as when it commences in an error of nomenclature.

In the chapter on the etiology of inflammation, our author follows closely the views of Paget, with occasional allusions to other well-known writers. We notice that he assigns longitudinal retraction of the elastic coat, as the cause of the dilatation of the arteries. This idea has the distinguished sanction of Mr. Paget; but it seems, nevertheless, insufficient to account for the phenomenon. Such an abbreviation of a capillary as to triple or quadruple its diameter does not in reality take place; and we are still disposed to assign at least a part of the distension to the *vis a tergo,* upon the paralyzed muscular coat.

The treatment of inflammation is introduced before the discussion of its sequelæ; an arrangement which renders it difficult to properly enforce those modifications of treatment, which may be called for by the tendencies of the process toward one or another termination. We may remark, here, in general terms, that Professor Smith's views of treatment are characterized by great attention to detail, and by neatness and nicety of application. The influence of such a quality upon the student is decidedly beneficial. The coarse, slovenly manner in which surgical remedies are too often applied, is a barrier to their success. The business prosperity of the surgeon depends very much upon his care in the application of a bandage, his precision of movement in reducing a fracture, or the accuracy of an incision; and, as the common people are very good judges of this kind of merit, it cannot be too strenuously inculcated by the teacher. It is, however, to some extent true, that the mind of the philosopher is incapable of the appreciation of detail, and to that fact must we assign the circumstance, that so many good investigators of the principles of surgery, are such bungling operators. Occasionally, we find a man born to be a surgeon, possessed of the philosophical mind, the resolute heart, the keen eye, the ready hand, and the unfailing nerve of the military genius, with the quick perception, and the gentle touch of a woman.

To return to the treatment of inflammation. Such paragraphs as that on the preservation of leeches, on page 64, are rather interesting to the apothecary than the surgeon; and belong of right in the U. S. Dispensatory. Those on their application, on the use of the seton, the moxa, or of issues, are neat and careful therapeutical descriptions, and well illustrated by plates. These matters of minor surgery, are apt to be neglected in our text-books, and we are glad to see their importance recognized.

Section 3, "On the Constitutional Treatment of Inflammation," is a most evident misnomer. It is an essay on the use and mode of application of the tools used in the treatment of inflammation. Why depletion cures inflammation, or whether it cures it, or whether it is proper to use it always, or only occasionally, are questions which do not seem to have suggested themselves to our author. On page 70, we have the "Treatment of Inflammation" condensed into a neat little wood-cut, showing "A. A Thumb Lancet," and "B. A Spring Lancet." On the opposite page, is a view of the operation of venesection, by "A. The Thumb Lancet;" while page 72 has an equally neat illustration of the manner of using "B. The Spring Lancet." In an operative surgery, under the head "Venæsectio," its exactness would have excited our admiration; but just at this place, we were studying how to cure inflammation, not how to pull the trigger of a spring lancet.

The same "error loci" is evinced in the subsequent paragraphs on Anodynes, where, instead of a discussion of the surgical efficacy of this class of remedies, we are told that opium is, when uncombined, a little apt to promote vascular activity, and that, in the treatment of inflammation, "one grain of calomel, and ten of Dover's Powder, given at night, is an excellent combination." This is a square-rule sort of treatment, the propriety of which is questionable.

Under the head of "Sedatives" we have a pet formula of aconitum napellus recommended as good for inflammation. We do not mean to be understood to assert that Professor Smith uses those words; but, dropping all badinage, we enter an earnest protest against this substitution of a chapter of materia medica for intelligent views of the treatment of inflammation. It is well known that, at this moment, the question whether the lancet shall or shall not be retained as the most familiar of weapons in combating inflammation, is a subject of active and able debate. The gradual growth in the medical mind of a distaste for active depletory measures finds no reflection in the chapter under notice, and, for aught it tells the student, he would be ignorant of the existence of any question as to the merits of depletion.

In the succeeding chapter, on the "Products of Inflammation," Professor Smith follows the usual division of the effusions consequent upon that process, viz., effusion of serum, of lymph, and of pus. But are not most writers in error here? Can an effusion of lymph occur without serum? Is there a power of selection in the vessels by which they choose the fibrinous element of the liquor sanguinis and effuse it, unaccompanied by the serum? Is a simple effusion of serum—the ordinary œdematous swelling—ever a direct immediate result of the inflammatory process proper? The straining through of the serum, which constitutes œdema, is a process of asthenia, not of sthenic disease. Only one effusion occurs in reality,—that of the liquor sanguinis as a totality. In this effusion variable quantities of fibrin may be found; this dependent, not on any selective power of the vessels, or any condition peculiar to inflammation, except the relative proportion of fibrin held in solution by the serum. If fibrin abounds, it will coagulate outside the vessels, forming a dense hard swelling; if it is wanting, we shall have a serous effusion, simply because the fibrin is not in the blood, and, it being wanting, one of the conditions of inflammation is also wanting. It seems to us that such a view of inflammatory effusions is at once truthful and simple. At present, the student almost necessarily imbibes wrong ideas of the constitution of the liquor sanguinis from the unnecessary distinction made by authors. Under the head of "effusion of pus" we shall recur to this idea.

The views given of the organization of lymph, and of union and adhesion, are those of Paget. We are pleased to see that the old dogma of the necessity of a degree of inflammation to secure a union is ignored, and that the student is taught to expect union without exudation. The description of the formation of fibro-cellular tissue by change of the lymph-corpuscle, and the formation of "cacoplastic lymph" in effusions having a deficiency of fibrin, are well given; as are those of the channelling process, and of the fusiform enlargement in the formation of new blood-vessels.

In denying the pus-secreting power of the "pyogenic membrane," Professor Smith virtually denies the effusion of pus. Like other recent authors, he speaks of pus as an "effusion," while he denies to its only source the power of effusing it. This bit of logic resolves itself into the conclusion that pus is never "effused" or secreted,—a conclusion which, by the way, is strictly correct. "Pus-secretions" and "pus-effusions" are misnomers which should be stricken from surgical etymology. The "pyogenic membrane" secretes lymph-corpuscles, and these are metamorphosed into pus. Metamorphosis is the word, and the sooner we come to it the better for the poor students, who with great labor acquire a knowledge of the effusion of pus, which must again be unlearned before they can obtain any correct notions. We have no doubt whatever that one-half the practitioners in the United States would be willing to swear that the pleura can become a pus-secreting membrane, and prove it by all the text-books on surgery, and by the lectures they heard at college. And yet that such is not the case, is a fact about which there can be no dispute, except with those unfortunate individuals whose ideas have been derived exclusively from one or other of these sources.

The description of "abscess," though a little cloudy, is in accordance with philosophical views. The "wall" of a pus-collection assumes its proper importance as an effusion of lymph in the areolar tissue, which may in time, or may not, assume the power of secreting lymph-corpuscles to undergo metamorphosis into pus. The assertion that, "whether acute or chronic, all abscesses have one common characteristic, to wit, the limitation of the space into which the pus is effused," is a little too broad, unless it is intended to distinguish diffused suppuration from abscess proper.

As to the treatment of abscess, we object with some personal feeling to that invention of the enemy known as spongiopiline. Its scalding, irritating, scratching, burning effects became fully known to us once when suffering from a slight dissection wound. The arrowroot poultice—not mentioned by our author—is the elysium of emollients.

Ulceration is treated of as "molecular death," in accordance with the modern view, although some question may exist as to how far our doctrine of cell-death, as taught to-day, differs from the theory of "disintegration," as taught in contradistinction to the views of Hunter, many years ago. We pass over the space devoted to the classification and treatment of ulcers, to come into the field of active surgical practice.

In the chapter devoted to Erysipelas, we find the following general rules of treatment:

"But generally the treatment of erysipelas should be directed almost entirely to the constitutional derangement, the local disturbance being only evidence, as a general rule, of constitutional derangement of an asthenic character. The treatment, therefore, should be based on this principle: Thus, in the first place, it is a good practice to administer an emetic, as it empties the stomach, gets rid of indigestible articles, and affects favorably the portal circulation, or, as the celebrated Dr. Rush used to say, in homely language, 'shakes the gall-bladder;' and, when followed by a dose of calomel and jalap, 'clears the ship fore and aft.'"

This general recommendation involves a sweeping hypothesis, and reminds us of a question put by a lawyer, the other day, to a succession of medical witnesses. The question covered three pages of foolscap, involving many different suppositions, but the doctors were compelled to answer yes or no. Now this plan of treatment supposes, 1. That the stomach should be emptied; 2. That it contains indigestible articles; 3. That the portal circulation must be disturbed; 4. That a shake to the gall-bladder is advisable; 5. That the ship must be cleared fore and aft. The tyro is not cautioned to be sure that the stomach contains anything wrong, or that there is any necessity for disturbing the secretion of bile; neither to inquire whether the "asthenic character" of the disease will be benefited by this knocking "fore and aft." Much better do we appreciate the talk about beef tea and quinine, which is suggested as an after-treatment, and we would have been glad to have seen some mention made of iron, in this connection, particularly the muriated tincture of that metal.

The subject of "Carcinoma," is handled in the manner most appropriate to a work of this kind. It is a brief *resumé* of the opinions of Paget, Lebert, Bennett, and others. Without attempting to discuss fully the pathological or microscopical characters of cancer, enough is said to give the student a fair idea of what constitutes cancer, and how much reliance may be placed on the presence of what is called the cancer-cell. Professor Smith does not define any special cell as peculiar to this disease; but following the more recent and sounder views of late observers, he assigns to it a variety of forms of cells; the multiplicity of forms constituting one of the signs. The recent discussion in the French Academy has had a salutary influence on our estimate of the value of the microscope in diagnosis; for, though many extravagant opinions were uttered, both for and against it, the general tone of the debate, while it lessens our confidence in the microscope alone, does not impair its real usefulness as an *assistant* in diagnosis. We may add that the chapter on carcinoma is, throughout, clearly written; and is an excellent, condensed account of the disease and its treatment; the latter striking us as sensible, and founded on just views of what may be accomplished.

In passing on we must express our regret that the section on animal poisons contains no mention of the valuable experiments of Professor Brainerd, of Chicago, whose widely-published essays on this subject have deservedly attracted so much attention. We shall have occasion to allude to the same apparent neglect of his countrymen in another connection.

Without stopping to notice the subject of wounds, other than to express our gratification at the large use made of Mayor's handkerchief bandages, we come to the subject of 'Fractures.'

Here we find that our author, who has so closely followed the pathology of

Paget heretofore, finds reason to differ on the matter of definitive and provisional callus. It will be recollected that but a short time since, Paget, in his lectures, advanced the opinion that "provisional" callus was not the beneficent plan of nature it had been supposed to be, but an unnecessary accident, depending upon inaccuracy in the adaptation of the broken fragments to each other. At the same time, or probably a little before, say four years since, Professor Hamilton, of Buffalo, promulgated entirely similar views. The question of priority in this doctrine, or at any rate in its publication in America, having been somewhat mooted, we may state from our positive recollection that Professor Hamilton's first publication preceded any announcement of Paget's theory in this country, of which we have any knowledge. We can hardly suppose Professor Smith to have been ignorant of this fact, it having been somewhat freely discussed in the journals, and were it not for the bad habit of some American authors of ignoring each other, and relying on European sources of opinion exclusively, we should doubtless have had some recognition of Professor Hamilton's fair claims to notice in this matter. We may add, too, that having carefully looked over the 130 pages devoted to fractures in this volume, we do not find Professor Hamilton's name mentioned in connection with the literature of fractures. We have no desire to claim undue respect for that gentleman, but we are entirely mistaken in our estimate of authorial merit, if the brief fracture-tables of Professor Hamilton do not entitle him to honorable mention from a systematic writer on that subject. We should insult Professor Smith by supposing that he is not familiar with them.

To return to provisional callus: Professor Smith prefers to adhere to Dupuytren, and consider this "ensheathing" callus (that is the preferable name) a necessity of fracture. He says: "Wherever a fractured bone is superficial, as in the clavicle or in the tibia, the bump or protuberance caused by the provisional callus, will often be too plain to be mistaken; whilst very many specimens of fractures recently united and surrounded by a true provisional callus, that is, one which is larger a few weeks after union than it is some months subsequently, are to be found in most surgical cabinets."

Admitting all this, we are at a loss to see what it proves. In our own practice, we have repeatedly, perhaps generally, in simple fractures of the tibia, secured a union without any perceptible callus. We attribute this to two causes: 1st. An accurate adaptation of the broken fragments, more easily attainable in the tibia than elsewhere, and, 2d. To comparatively slight separation of the periosteum from the bone. Supposing the periosteum to be lifted from the bone to any considerable extent, the clot of blood intervening will inevitably organize into bony tissue; but, the periosteum remaining attached, any deposit of bone taking place in it, will be due to inflammatory exudation. This inflammatory exudation may be avoided by early and accurate adjustment of the fragments. Ensheathing callus, found in museum specimens, only proves a faulty adaptation or extensive lesion of the periosteum; but even where such extensive lesion occurs, should the periosteum regain its union with the bone by first intention, no callus would, in our opinion, occur. These views are analogically supported by what we daily see in union of the soft parts. Furthermore, wherever we have found difficulty in maintaining the adjustment of the fracture, we have also found abundant provisional callus.

We do not find that any mention is made by our author of the delay in adjustment of fractures, which has recently become fashionable. He assumes, by this failure to notice it, that a fracture should be dressed at the earliest possible time, and in a manner intended to be permanent. We are glad to find that a text-book intended for students, thus ignores a practice which is the *ne plus ultra* of blind confidence in nature. It is assumed that it is as well to wait until, inflammation having subsided, the process of union can begin. But when will inflammation subside, with a fragment of bone irritating the soft parts? Will not an extensive ensheathing callus result from the failure to bring the periosteum back to the bone? Viewed in the light of all we know of union of tissues, this new-fangled notion is one of the most absurd in the history of surgery.

We are surprised that the relations of the middle meningeal artery to fractures of the lower anterior angle of the parietal bone, are not more fully mentioned. During the past summer we have been twice called upon to make autopsies, which revealed simple fissure of the bone at this point, but involving a rupture of the artery at the part where it is bridged by bone, and causing death by effusion of blood.

For fractures of the clavicle, Fox's apparatus is evidently the favorite of our author. We are inclined to consider it the best in use, but we should dissent from the opinion expressed, that a perfect result is attainable with it at will. Of 53 cases of this fracture recorded by Professor Hamilton,* 13 are mentioned as treated by Fox's apparatus. Of these, 7 are reported imperfect, 5 slightly imperfect, and only 1 perfect. Many surgeons will recollect the discussion on this point at the meeting of the American Medical Association in Philadelphia, in 1855, and the result of an examination of cases under treatment at the Pennsylvania Hospital. Moreover, the opinion of all surgeons concedes the difficulty of securing a positively perfect result in fractures of the clavicle; and so long as our text-books do not openly recognize this opinion, so long will practitioners and patients alike expect too much, and mal-practice suits result. We are not of those

* Trans. of Amer. Med. Association, 1855, page 418.

who would depreciate the triumphs of surgery; but mal-practice suits are abundant enough to teach us that it is an expensive luxury to lay claim to skill which we do not possess.

The notice of fractures of the scapula, is judiciously brief. We are sorry to see that impossible wood-cut reproduced from Fergusson. Fractures of the coracoid process are mentioned as very rare. Compared with other fractures of the scapula, we think their frequency is under-estimated. As to fractures of the acromion, our author raises the hypothesis that they are frequently only separations of the epiphysis, it having failed to co-ossify fully. Dr. J. B. S. Jackson, of Boston, is, we believe, the author of this suggestion or hypothesis.

Fractures of the humerus are more fully noticed, but we find no mention of the recent proposition to dress with the elbow extended, thus securing the weight of the forearm as an extending power, and materially diminishing the movement of the fragments upon each other, which is so common a cause of non-union in this bone. A glance will show that this form of dressing, transfers all such motion to the shoulder-joint, while the flexed position makes every motion felt at the point of fracture, the lower segment describing the arc of a circle.

Fractures of the forearm are well and practically described; Barton's fracture especially. Bond's splint is described and recommended. Probably the author was not aware that Bond's splint has been recently very much improved by putting in a joint to correspond with the wrist; so that any degree of lateral flexion may be obtained, while the same splint is applicable to either arm.

So far as other fractures are concerned, we find little that is noteworthy. The descriptions of symptoms, the differential diagnosis, and the treatment, are given with, we believe, uniform correctness and clearness.

In the treatment of ununited fracture, the author's natural preference for his own mode of treatment leads him into some injustice to Professor Brainerd. Professor B. has made most elaborate and practical experiments upon the management of this accident, and it is unfair to him to pass by his labors with the single sentence, "Dr. Brainerd, of Chicago, has also proposed to drill a number of holes in the end of a bone with an awl, with the same view,"—that is, the production of inflammation. Dr. Brainerd has not only proposed this operation, but has successfully performed it in a series of cases sufficient to entitle it to a respectful consideration. Other surgeons have adopted it, and without any of those dangerous results which are described on page 449. We quote:

"But in order to make use of any of these modes of treatment, it becomes necessary that the patient should for a long time retain the recumbent position; whilst he is exposed to the risks of the creation of a compound fracture, in which suppuration may take place, and where the pus travelling up and down the limb in the intermuscular cellular substance, will establish a drain upon his vital energies, which may cost him his life. Or, if these dangers are escaped, erysipelas may be set up, and he will be exposed to all its evils. It has also happened more than once, that, after submitting to one of these painful operations, and perilling his life, he has subsequently been obliged to submit to amputation."

Now let us inquire how much of this pathetic picture belongs to Brainerd's operation, and how much to Physic's and Dieffenbach's, with which it is coupled. The confinement to the bed is only necessary in fractures of the lower extremity; the risks of compound fracture are beyond our comprehension; we are at a loss to see how an operation like Brainerd's can produce the suppuration which has such terrible consequences; and we have yet to hear of any erysipelas resulting from it. All these disasters, with the amputation which closes the tearful catalogue, are the results of Dieffenbach's or Physic's operations; and it is in the highest degree unfair to couple Brainerd's operation with them, so as to make a nominally truthful paragraph convey an erroneous impression. We regret to speak with so much plainness; but the matter required notice, and Dr. Brainerd deserves a vindication.

As to Professor Smith's dressing, it consists of an apparatus forming a firm casing for the affected limb, so as in fact to constitute an artificial limb. The idea is philosophical, and, we should judge, likely to be successful in many cases. The time occupied in the cure is a serious objection. In some cases, recovery has occurred so soon as six weeks; in others, seventeen, nineteen weeks, or five months were required. Compared with Dr. Brainerd's operation, the latter has the merit of greater cheapness, less incumbrance with splints, and more positive certainty. But the two together may be considered as essential improvements, which will do away with the necessity of amputation.

Reid's method of reducing dislocations of the hip-joint is fully indorsed. The laughable dispute as to the originator of this operation receives only casual notice. We have a friend who is extremely indignant towards Dr. Kane for claiming to discover the open Polar Sea, when he (our friend) had long been aware of its existence as a matter of theory. Similar discoverers have magnified their own knowledge of this method of reduction. It is something incomprehensible that they were so continent in their knowledge.

The treatment recommended for *morbus coxarius* is virtually that of Dr. Physic. Perfect rest and confinement on a splint are enjoined from the outset; the use of purgatives, bleeding, and counter-irritants form its principal features.

We are told in reference to the use of cathartics in this affection:

"This purging not only acts on the principle of revulsion, but also by relieving the congestion of the portal circle. The function of nutrition is, therefore, more actively carried on, and instead of being exhausted by frequent purgation, the patient sometimes actually grows fat under the treatment."

Here is another specimen of reasoning from hypothesis. When has it been proved that congestion of the portal circle is one of the conditions of hip disease? We had hoped that the strumous character of this affection was recognized. Is it possible that we have made no advance during the last half-century? Are the results obtained from this mode of treatment such as to encourage us? The first element in the treatment of morbus coxarius is to invigorate the general health. Out-door air, moderate exercise, tonics, and nutritious diet from the outset, and the avoidance of all debilitating agents, are the means to be relied upon for this purpose. A treatment which destroys appetite, wastes the muscles, and enfeebles the powers of life, will never get rid of the constitutional vice which *is* morbus coxarius. How to reconcile the necessity of rest to the afflicted joint with a proper maintenance of the general health is the problem which now demands the ingenuity of the surgeon.

The space assigned us is already occupied. We can only notice the excess of brevity, which marks the description of the diseases of the eyeball and its appendages. It is better to leave the eye to special writers, than to waste time upon entirely inadequate descriptions.

As a whole, we should speak of Smith's Practice of Surgery, as a doubtful addition to our means of teaching. The practitioner, seeking for that which is new, will find but little to reward his quest; neither will he find it a volume which marks the present state of surgical science. But, in by far the greater proportion of its teachings, he will find the word of assured truth—conservative to a fault—but reliable. The reader, interested in surgical discussions and in the claims of American surgeons to honorable mention, will peruse this volume with pain. There is the old subservience to European opinion, the old colonialism, which eighty years of national existence has not destroyed. He will notice, too, occasionally, a want of recognition of the labors of his compeers, which, whether it originates in the subservience to European mind, already noticed, or, in other reasons, is alike worthy of censure.

But we have devoted sufficient space to detail, to render it unnecessary to give any formal opinion of the work under notice. When we sat down to its perusal, we expected the pleasure of conceding to it, in all departments, a high degree of merit. Wherever we have been able, difficult as was the task, we have done so. In our meed of praise or censure, we have endeavored to be alike truthful.

We have, however, omitted to mention a remarkable inequality, which is evident throughout the volume; because we were unable to explain it. Haste in the preparation, a strong but unenlightened deference to Paget in all matters of pathology, and the attempt to write down to the comprehension of students, are the probable causes of this inequality. Whatever its source may be, we trust, that if Professor Smith is to take a permanent place among our surgical writers, which, however, we are inclined to doubt, he will avoid a similar fault in the future.

